# Atypically slow processing of faces and non-faces in older autistic adults

**DOI:** 10.1177/13623613211065297

**Published:** 2021-12-28

**Authors:** Joe Bathelt, P Cédric MP Koolschijn, Hilde M Geurts

**Affiliations:** 1 University of Amsterdam, The Netherlands; 2 Royal Holloway, University of London, UK; 3 Leo Kannerhuis, Youz/Parnassiagroup, The Netherlands

**Keywords:** aging, autism spectrum disorder, face processing, functional magnetic resonance imaging, reaction time

## Abstract

Face recognition is a fundamental function that requires holistic processing. Differences in face processing have been consistently identified in autistic children, but it is unknown whether these differences persist across the adult lifespan. Using event-related functional magnetic resonance imaging, we measured holistic face processing with a rapid Mooney faces task in 50 autistic and 49 non-autistic participants (30–74 years). Behavioral tasks included a self-paced version of the same paradigm and a global–local processing task (Navon). Reduced detection rates for faces, but not non-faces, were found in autistic adults, including slower responses on all conditions. Without time constraints, differences in accuracy disappeared between groups, although reaction times in correctly identifying faces remained higher in autistic adults. The functional magnetic resonance imaging results showed lower activation in the left and right superior frontal gyrus in the autism group but no age-related differences. Overall, our findings point toward slower information processing speed rather than a face recognition deficit in autistic adults. This suggests that face-processing differences are not a core feature of autism across the adult lifespan.

**Lay abstract:**

Some theories suggested that social difficulties in autism arise from differences in the processing of faces. If face-processing difficulties are central to autism, then they should be as persistent as social difficulties across the lifespan. We tested this by asking autistic and neurotypical participants between 30 and 75 years to complete face detection tasks. Both autistic and neurotypical adults responded more slowly with age. When participants had to respond quickly, autistic adults made more errors in face detection regardless of their age. However, when the time constraint was removed, autistic adults performed as well as the neurotypical group. Across tasks, autistic adults responded more slowly when asked to detect both face and non-face stimuli. We also investigated brain activation differences in the face detection task with functional magnetic resonance imaging. The results indicated lower activation in the autism group in the left and right superior frontal gyrus. The superior frontal gyrus is not typically implicated in face processing but in more general processing, for example, keeping instructions in mind and following them. Together with the behavioral results, this suggests that there is no specific deficit in face processing in autistic adults between 30 and 75 years. Instead, the results suggest differences in general processing, particularly in the speed of processing. However, this needs to be investigated further with methods that are more sensitive to the timing of brain activation.

## Introduction

Autism spectrum disorder (ASD)^1^ is a neurodevelopmental condition that is typically diagnosed in childhood but that exerts a lifelong influence. Differences in social processing and interaction are among the most prominent behavioral features of autism (American Psychiatric Association, 2000). These differences persist across the adult lifespan (Howlin et al., 2013). Furthermore, lower quality of life related to social function remains one of the major concerns from childhood until old age (van Heijst & Geurts, 2015). The etiology of these difficulties is yet unknown, but genetic research suggests a strong contribution from neurobiological mechanisms. Atypical face processing has been suggested as a potential pathway that could lead to the social features of autism (Laycock et al., 2020), as many aspects of successful social interaction involve processing information from faces.

Indeed, faces are complex visual stimuli that require specific processing. Unlike other types of visual processing (e.g. objects), face processing requires a holistic or configurational rather than a part-based processing strategy (Maurer et al., 2002; Piepers & Robbins, 2012). Holistic processing is thought to be particularly important for fast and accurate face recognition (Richler et al., 2011; Wang et al., 2012). Moreover, in typical development, there is support for a close association between face perception and precedence of global over local processing (Darling et al., 2009; Martin & Macrae, 2010; Weston & Perfect, 2005). Furthermore, recent meta-analyses indicate that autistic individuals are slower in global-order perception (Van der Hallen et al., 2015) and display a bias toward local processing (Muth et al., 2014). While these results provide strong evidence of differences in face processing in autistic people, the findings are limited to children and young adults (e.g. Dawson et al., 2002; Gepner et al., 1996; Klin et al., 1999; Sigman et al., 1992). If differences in holistic face processing are a core aspect of being autistic, then these differences should be present across the lifespan, in parallel with social communication difficulties. Here, we redress this gap by investigating differences in holistic face processing in autistic adults (30–75 years).

**Figure 1.**
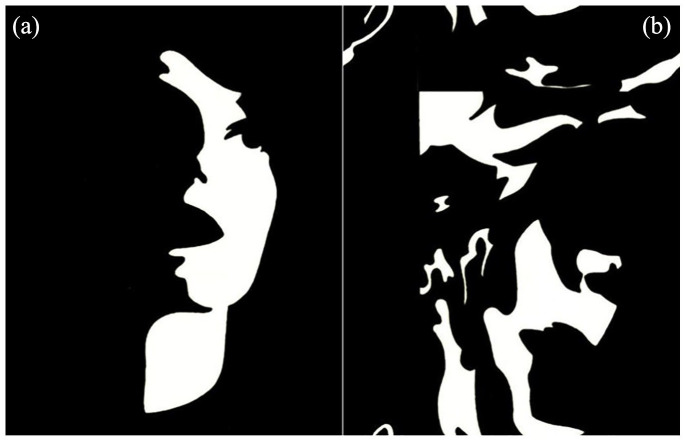
Mooney face stimuli used in- and outside scanner task. (a) Upright Mooney face and (b) scrambled Mooney face (i.e. non-face).

Specifically, we used functional magnetic resonance imaging (fMRI) to examine brain activation patterns in autistic adults (total *N* = 99; ages 30–75 years) while performing a rapid perceptual-closure face recognition paradigm (Grutzner et al., 2010; Sun et al., 2012). The term “perceptual closure” refers to the ability to form a global and coherent perceptual representation on the basis of few (local) details (Mooney & Ferguson, 1951). A classic example is provided by two-tone (black and white) images of human faces (Mooney faces), derived from photographs of faces under asymmetrical lighting conditions (Mooney, 1957; Mooney & Ferguson, 1951). An important aspect of Mooney stimuli is that most local and relational information has been concealed, because all luminance values are transformed to either black or white. However, the stimuli can still be perceived as faces through holistic processing, even though they do not include face parts that are recognizable in isolation (Figure 1; Latinus & Taylor, 2005, 2006). Importantly, evidence from fMRI studies indicates that the perception of Mooney faces and real faces results in similar brain activation patterns (including the fusiform gyrus; e.g. Andrews & Schluppeck, 2004; Grutzner et al., 2010; Kanwisher et al., 1998; McKeeff & Tong, 2007). Based on previous studies, we expected reduced detection rates for faces and elevated reaction times (RTs) in the ASD group (Griffin et al., 2021; Naumann et al., 2018; Rouse et al., 2004; Sun et al., 2012). Furthermore, we hypothesized atypical activation patterns in response to faces, primarily in occipital and temporal regions based on fMRI meta-analytical evidence (Samson et al., 2012).

We assessed autistic participants across a broad age range to ascertain if there are age-related differences in holistic face processing across the adult lifespan. Prior research has suggested that there is no age-related decline in holistic face-processing capabilities until older age (Boutet et al., 2015; Daniel & Bentin, 2012; Meinhardt-Injac et al., 2014). For instance, performance on Mooney face detection was found to be stable between ages 15 and 65 years after which performance started to decline (Carbon et al., 2013). This relatively stable trajectory across the lifespan may suggest that possible impairments in holistic processing in autistic adults will also be relatively stable with increasing age. Thus, we expect no age-related differences in performance or brain activity in our sample.

To aid the interpretation of our findings, we tested if differences in global–local processing could potentially explain face-processing differences in autistic individuals (Davies et al., 1994; Gross, 2005; Wallace et al., 2008; Weigelt et al., 2012). To this end, we included a global–local processing task (Navon hierarchical letters; Navon, 1977). Based on meta-analytical findings (Van der Hallen et al., 2015), we hypothesized that autistic individuals would be slower in the global processing condition, and show faster or similar speed in the local condition compared to the typical comparison group. We also expected positive associations between performance and RTs between the face-processing Mooney task and the local–global processing Navon task. In sum, the aim of this study was to test if holistic face recognition skills are impaired in autistic adults and if holistic face recognition skills differ across the adult lifespan in autism.

## Methods

### Participants

A total of 50 subjects with an ASD diagnosis and 49 controls (CTRL) between 30 and 75 years were recruited from a cohort of participants of a large-scale behavioral study (“Autism and Aging,” NWO VIDI 452-10-003). Participants were recruited via advertisements, our website, and personal contacts and pre-selected for the MRI-study based on willingness and absence of MRI-contraindications. We used the following diagnostic inclusion criteria for ASD participants: (1) a formal pre-existing diagnosis of ASD from a specialized clinic^2^ and (2) confirmation of diagnosis based on research criteria by an Autism Diagnostic Observation Schedule module (ADOS) module 4 trained psychologist: 33 individuals had a score above the cut-off of the ADOS (>7; Lord et al., 1989) and those not scoring above this cut-off did score above the AQ cut-off (>26; Autism-Spectrum quotient, 50-item list, Koolschijn et al., 2017).

All participants had an estimated intelligence quotient (IQ) above 80 based on two subtests of the Wechsler Adult Intelligence Scale–Fourth Edition (WAIS-IV; Wechsler, 1981). There were no between-group differences for IQ, age, sex, and handedness (Table 1). Participants had no self-reported history of neurological disorders, chronic illness, learning disabilities, or schizophrenia. For the control (CTRL) group, an additional exclusion criterion was a first- or second-degree family member with ASD. Participants gave written informed consent for the study and received fixed payment for participation and travel reimbursement. The internal review board at the University of Amsterdam (#2013-PN-2668).

**Table 1.** Demographic variables.

Description	ASD^a^ (*N* = 50)	CTRL (*N* = 49)	Statistics
Number of males (%)	34 (68%)	32 (65%)	*χ*^2^ = 0.81, *p* = 0.776
Age in years (SD) [range]	51.02 (12.34) [30.04–73.98]	50.14 (11.94) [30.62–73.77]	*F* = 0.13, *p* = 0.719
IQ (SD) [range]	116.26 (16.37) [86–155]	111.59 (15.78) [80–141]	*F* = 2.09, *p* = 0.152
MMSE total score (SD) [range]	29.18 (0.96) [27–30]	28.98 (1.11) [26–30]	*F* = 0.924, *p* = 0.339
Level of educational attainment^b^	1/15/22/12	1/11/27/10	*χ*^2^ = 1.30, *p* = 0.73
Handedness			*χ*^2^ = 1.01, *p* = 0.951
Left	5	4	
Right	42	42	
Ambidexter	3	3	
Age first diagnosis (years)	45.45 (13.61) [11.22–68.04]	*N.A.*	
ADOS total	7.98 (3.33) [1–19]	*N.A.*	
Language and communication	2.58 (1.36) [0–5]	*N.A.*	
Social reciprocity	5.4 (2.49) [1–14]	*N.A.*	
Fantasy	1.12 (.52) [0–2]	*N.A.*	
Restricted and repetitive behaviors	0.3 (0.58) [0–2]	*N.A.*	
ADOS cut-off (<7)^c^	17 (43%)	*N.A.*	
AQ total	36.26 (6.58) [19–47]	12.98 (5.89) [4–26]	***F* = 349.98, *p* < 0.001**
AQ cut-off (<26 ASD, >23 CTRL)	4 (8%)	0	***χ*** ^2^ ** = 84.21, *p* < 0.001**
Medication *N* (%)	40 (80%)	19 (39%)	***χ***^2^ **=** **17.47, *p*** **<** **0.001**
Antidepressants	15 (43%)	2 (4%)	***χ***^2^ **=** **11.69, *p*** **=** **0.001**
Antipsychotics	8 (16%)	0	***χ***^2^ **=** **8.53, *p*** **=** **0.003**
Sedatives	7 (14%)	0	***χ***^2^ **=** **7.38, *p*** **=** **0.007**
Stimulants	6 (12%)	0	***χ***^2^ **=** **6.26, *p*** **=** **0.012**
Antiepileptics	2 (4%)	1 (2%)	*χ*^2^ = 0.32, *p* = 0.57
Antiparkinson	1 (2%)	0	*χ*^2^ = 0.99, *p* = 0.32
Migraine	4 (8%)	0	***χ***^2^ **=** **4.09, *p*** **=** **0.043**
Non-psychotropic medication^d^	26 (52%)	17 (35%)	*χ*^2^ = 3.02, *p* = 0.082
fMRI movement			
DVARS (%)	1.19 (0.091)	1.18 (0.093)	*t* = 0.26, *p* = 0.789
FD mean (mm)	0.27 (0.114)	0.27 (0.107)	*t* = 0.01, *p* = 0.989

ASD: autism spectrum disorder; CTRL: control; SD: standard deviation; IQ: intelligence quotient; MMSE: mini-mental state examination; N.A.: not applicable; ADOS: Autism Diagnostic Observation Schedule; AQ: autism-spectrum quotient.

Numbers in bold reflect significant between-group differences.

a For the ADOS-only group (above cut-off >7), there were no differences in demographics compared to controls: *N* = 33 (23 male), mean age: 48.77 (11.50) [33.04–70.84], all *p*’s > 0.09, except for AQ score *F* = 277.12, *p* < 0.001.

b The numbers between the slashes indicate the number of participants who had pre-vocational education/junior general secondary or vocation education/senior general secondary education or vocation colleges/university education based on the Verhage scale (Verhage, 1964).

c All subjects below threshold scores on the ADOS, had scores *above* the clinical cut-off for the AQ.

d Includes a.o.: blood pressure/thinner, antihistamines, cholesterol, sleeplessness, asthma, heartburn, and diabetes.

### Data acquisition

All participants were scanned on a 3-Tesla whole body Philips Achieva MRI system (Best, The Netherlands). Functional data were acquired using a single shot GE-EPI sequence during two functional runs of 213 volumes each, of which the first 4 volumes were discarded to allow for equilibration of T1 saturation effects (TR = 2 s), echo time = 27.63 ms, 37 slices of 3 mm × 3 mm × 3 mm (slice gap = 0.3 mm), field of view (FOV) 240 mm^2^, and 80 × 80 matrix. Two high-resolution T1-weighted anatomical scans were obtained: 3DFFE, multi-shot turbo field echo (TFE): TR = 8.2 ms; TE = 3.8 ms, 220 slices, voxel size = 1 × 1 × 1 mm^3^, FOV = 240 × 188, matrix = 80, and 2D SENSE: P(RL) = 2.5 and S(FH) = 2. Head motion was restricted using a pillow and foam inserts that surrounded the head. Visual stimuli were projected onto a screen that was viewed through a mirror.

### Community involvement

Four autistic adults are part of the study advisory committee for the overarching study (Geurts et al., 2021). However, for this specific subproject within the larger study, there was no explicit and formalized community involvement.

### Procedure and experimental design

#### Mooney task

All participants were tested individually and were trained to lie still in a mock scanner, which simulated the environment and sounds of an actual MRI scanner. Details of the Mooney task have been published previously. In short, we used the same Mooney paradigm and stimuli as described in Grutzner et al. (2010) and Sun et al. (2012). Participants performed a Face/Non-Face detection task using Mooney Faces presented upright (Faces) and scrambled Mooney faces (i.e. Non-Faces; see Figure 1). Before scanning, participants performed a practice block of 10 trials to familiarize them with the speed of the task. During scanning, participants were presented with a random sequence of upright and inverted-scrambled stimuli displayed in the center of a translucent screen, which were shown for 200 ms. Using short stimulus presentation times (200 ms), we aim to rule out utilization of different strategies (e.g. heuristics), and overcome possible influences of abnormal gaze patterns (Falck-Ytter & von Hofsten, 2011). Participants performed two runs of the task, consisting of 50 trials of each condition per run. The inter-stimulus interval ranged between 3500 and 4500 ms and a fixation cross was presented in the center of the screen between trials. Participants responded as fast as possible with a button press (left/right index finger) to both Face and Non-Face stimuli and hand assignment was counterbalanced across participants.

After scanning, participants performed the Mooney task again, but in a self-paced version. We added this variant to test whether participants with ASD are capable of adequately performing this task without time constraints and whether timing differences would lead to improved performance. All stimulus presentation was controlled using the Presentation software package (Neurobehavioral Systems). For the in-scanner behavior and post-testing, mean accuracy and RTs as a function of stimulus type were analyzed, including the discrimination index A (Grier, 1971; Stanislaw & Todorov, 1999).

#### Navon task

**Figure 2.**
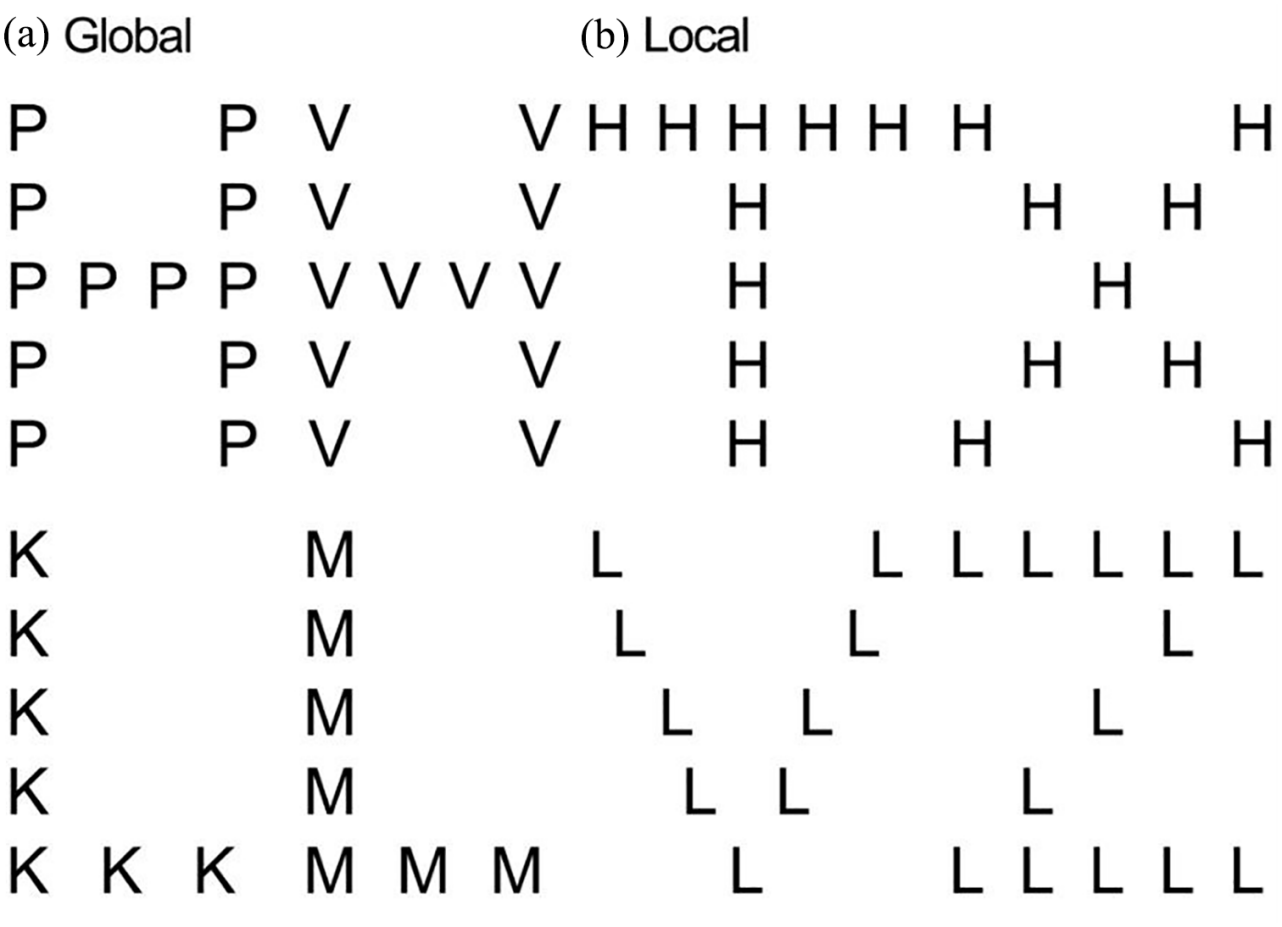
Navon hierarchical letters task. For the H-target, compound letters consisted of a number of small capital P’s or V’s to form a global H (global condition (a)) or small capital H’s to form either a global X or T (local condition (b)). For the L-target, compound letters consisted of a number of small capital K’s or M’s to form a global L (global condition (a)) or small capital L’s to form either a global Z or V (local condition (b)). Only one stimulus was presented at a time.

A Navon hierarchical letter task (Navon, 1977, 1981) was administered after scanning. We utilized a modified version of the Navon task, where participants were instructed to identify a target letter (H or L) regardless of whether it occurred on the local or global level. For each trial, a target letter occurred randomly at the global or local level. One stimulus was presented at a time. The task required participants to search and focus on global or local Navon stimuli while ignoring distractors at the other level (see Figure 2).

In the Navon task, participants were required to respond to the target letters (irrespective of modality) using their left and right index fingers corresponding to two keyboard buttons. All letters appeared in black Calibri font (size 28 for local letters) on a white background, with a viewing distance of approximately 40 cm, unrestrained. The stimulus remained on the screen until the participant had made a response, with a 250–500 ms interval between trials.

The task consisted of an introduction block of 10 trials to test whether participants understood the task, followed by two blocks of 150 trials each in which global and local trials were presented randomly. Mean global and local RTs were calculated for each participant, including local–global precedence (local RT–global RT).

### Data analysis

#### Behavior

Data were checked for outliers using outlier labeling (Hoaglin et al., 1986; Hoaglin & Iglewicz, 1987). Normality of the data was checked by running the Shapiro–Wilk test. Depending on the outcome of these analyses, linear regression or a non-parametric Mann–Whitney *U* tests for two independent samples were performed to test our hypotheses of better and faster performance on face recognition in controls compared to ASD.

Analyses started with assessment of bivariate relations, that is, correlations, between outcome variables of both conditions (accuracy, mean overall RT and RTs with accuracy, and discrimination index) and our predictors. We distinguished two types of predictors: our predictors of interest to test whether there were group and age-related differences in performance (group, age, and group-by-age-interaction), which were not included in the correlational analyses, and possible predictors that were included (sex, estimated IQ, handedness, and AQ score if significant subscales were also tested). For example, if sex had been identified as a possible covarying variable, that is, found to be associated with an outcome variable, it would be included in the subsequent regression analyses. For the regression analyses, Cook’s distances and the distribution of the residuals were inspected to identify influential data points. If such cases were identified, analyses were repeated excluding these cases.

Second, to study whether earlier inconsistent findings in Mooney faces paradigms could be related to differences in task speed (i.e. rapid vs self-paced), we analyzed accuracy and RTs of the outside scanner version and additionally compared those to in-scanner performance. For the Navon task, we subjected accuracy, RTs, and post-error/correct RTs (RTs after an incorrect/correct response) for each condition, and local–global precedence to one-way analyses of variance (ANOVAs) with group as a between factor. Third, to investigate the interrelations between holistic face processing and global–local processing, bivariate correlations were calculated and compared between groups using Fisher’s *r*-to-*z* transformations.

#### fMRI data analysis

Here, we tested whether the underlying neural signature of holistic face recognition skills in autistic adults is atypical. The pre-processing was performed using *fMRIPrep* 20.1.1 (Esteban et al. (2018, 2019); RRID:SCR_016216), which is based on *Nipype* 1.5.0 (Gorgolewski et al. (2011, 2018); RRID:SCR_002502). A detailed description of the pre-processing steps is provided in the Supplementary Materials.

Pre-processed data were analyzed using Nilearn v0.7.0 (PRID:SCR_001362). Presentations of faces and non-faces with correct and incorrect responses were used as explanatory variables and were convolved with the hemodynamic response function (HRF) proposed by Glover (1999). Furthermore, the motion parameters and their temporal derivatives estimated during pre-processing stage were included as confound regressors (Siegel et al., 2014). In addition, a binarized motion outlier regressor was used that flagged volumes that exceeded a threshold of 0.5 mm FD or 1.5 standardized DVARS. Spatial smoothing was performed with a full-width half-maximum (FWHM) Gaussian kernel of 5 mm. A gray-matter mask based on the ICBM152 template was used for all statistical analyses. Between-subject analyses focused on the contrasts of interest, that is, Face (correct) > Face (incorrect) and Face (correct) > Non-face (correct). We estimated the effect of group (ASD vs CTRL) and the interaction between group and age. We ran additional control analyses to estimate the effect of group when including age as a nuisance regressor.

## Results

### Behavior

#### Mooney faces

Three participants (two ASD and one CTRL) were identified as outliers with respect to overall hit-rate based on outlier labeling (Hoaglin et al., 1986; Hoaglin & Iglewicz, 1987) and were excluded from further analyses (behavioral and fMRI). All variables were normally distributed, except for accuracy (ASD: *W* > 0.8, *p*’s < 0.024; CTRL: *W* > 0.87, *p* < 0.021) and RT in the Face condition (ASD: *W* = 0.93, *p* = 0.007; CTRL: *W* = 0.92, *p* = 0.002) of the scanner task. Across both groups, there were more correct trials in the Non-Face compared to the Face condition (Face: M = 71.697, SE = 1.5397, range: 4–94; Non-face: M = 82.939, range: 7–100; SE = 1.7885; paired *t*-test: *t*(98) = −51.4, *p* < 0.001, (*n* trials)). However, there was no difference between stimulus types in RT variability indicating that the conditions were comparable (Face: M = 182.55, SE = 7.691; Non-Face: M = 185.949, SE = 7.843; 2 × 2 ANOVA with condition and response: *F*(1, 380) = 0.227, *p* = 0.634, (ms)).

Our first aim was to test whether holistic face-processing skills are impaired in autistic adults. Table 2 shows the results of in-scanner and post-test behavioral performance. Controls performed slightly better in the Face condition than ASD (76.85% vs 71.14%, *U* = 864, *Z* = −2.1, *p* = 0.035), and were faster in all conditions, irrespective of accuracy (all *p* < 0.05; Table 2A). Importantly, using repeated measure ANOVA models to test for accuracy and RT differences between runs, we demonstrate no learning effects for this paradigm (for both groups: all *p* > 0.8). Our second aim involved testing differences between rapid versus self-paced versions of our Mooney paradigm to test if differences can be attributed to processing speed. In the outside scanner task without time constraints, differences in accuracy disappeared between groups, although RTs in correctly identifying Faces remained higher in ASD compared to controls (*F* = 6.13, *p* = 0.015; Table 2B).

**Table 2.** Behavioral performance Mooney faces in scanner and post-test.

	ASD (*N* = 48)	CTRL (*N* = 48)	Statistics
A. Scanner			
Accuracy % correct (SD) [range]			
Face	71.14 (13.30) [35–93]	76.85 (9.97) [51.14–94]	***U* = 864, *Z* = −2.1, *p* = 0.035**
Non-Face	85.88 (15.30) [30–100]	85.75 (13.40) [42–100]	*U* = 1102, *Z* = −0.36, *p* = 0.717
RT in ms (SD) [range]			
Face	738.9 (173.6) [320.2–1201.9]	616.3 (91.4) [322.7–842.5]	***U* = 584, *Z* = −4.16, *p* < 0.001**
Correct	726.6 (171.1) [323.4–1156.7]	596.0 (85.6) [321.9–803.4]	***U* = 569, *Z* = −4.27, *p* < 0.001**
Incorrect	866.5 (246.1) [335.0–1590.0]	743.1 (157.6) [324.1–1302.3]	***U* = 794, *Z* = −2.62, *p* = 0.009**
Non-Face	776.7 (193.1) [303.6–1422.7]	693.3 (133.6) [341.8–1211.4]	***F* = 6.06, *p* = 0.006**
Correct	788.4 (182.8) [309.9–1353.2]	706.0 (135.1) [384.1–1232.9]	***F* = 6.32, *p* = 0.014**
Incorrect	911.1 (318.5) [299.7–2047.9]	691.6 (174.9) [311.1–1150.4]	***F* = 16.97, *p* < 0.001**
Discrimination index	0.86 (0.08) [0.59–0.96]	0.88 (0.07) [0.57–0.95]	*F* = 2.70, *p* = 0.104
B. Post-test			
Accuracy % correct (SD) [range]			
Face	77.9 (19.9) [30–100]	80 (20.7) [30–100]	*F* = 0.252, *p* = 0.617
Non-Face	96.3 (8.41) [60–100]	95.6 (7.41) [70–100]	*F* = 0.149, *p* = 0.700
RT in ms (SD) [range]			
Face			
Correct	1592.9 (1394.3) [415.7–6517.1]	1059.7 (532.5) [475.3–2927.2]	***F* = 6.13, *p* = 0.015**
Incorrect	4655.4 (14,003.3) [410.5–87,948]	2889.0 (2466.2) (575–11,708)	*F* = 0.526, *p* = 471
Non-Face			
Correct	2176.2 (1984.1) [450.7–10,606.9]	2083.5 (1343.2) [557.8–6163.9]	*F* = 0.072, *p* = 0.789
Incorrect	4262.5 (7105.2) [400–25,142]	4126.1 (3970.7) [567.5–13,636]	*F* = 0.004, *F* = 951

ASD: autism spectrum disorder; CTRL: control; SD: standard deviation; RT: reaction time.

Numbers in bold reflect significant between-group differences. Outliers are not included in these analyses.

In exploratory paired-sample *t*-tests, we examined the improvement of accuracy between in- and outside scanner performance. Individuals with ASD, but not controls, performed significantly better in the Face condition of the self-paced version compared to in-scanner performance (ASD: *t*_(47)_ = 2.59, *p* = 0.013, mean-percentage difference = 11.7%; CTRL: *t*_(47)_ = 1.34, *p* = 0.185, mean percentage difference = 3.4%). Both groups improved in the Non-Face condition (ASD: *t*_(47)_ = 4.55, *p* < 0.001, mean percentage difference = 18.2%; CTRL: *t*_(47)_ = 4.66, *p* < 0.001, mean percentage difference = 15.2%).

#### Regression analyses of moderator variables

To test whether moderator variables, including our variables of interest (age, and group-by-age interaction), were related to differences in face recognition performance, we performed regression analyses. We started with correlations between all variables of interest (Table 3) to identify potential moderators. We found significant correlations between age and AQ scores with accuracy and RTs. Furthermore, we observed medium-size correlations between AQ subscales and accuracy in both the face and non-face condition (see Table 3). These variables were then included in the subsequent regression analyses for the dependent variables.

**Table 3.** Bivariate associations between Mooney faces outcome measures and demographic variables of interest.

Variable																			
% FaceCor																			
% Non-FaceCor	−0.292**																		
Post-test % FaceCor	0.572**	0.151																	
Post-test % Non-FaceCor	−0.166	0.251*	−0.037																
MRT Face	−0.253*	0.118	−0.325**	0.134															
MRT Non-Face	0.157	−0.182	−0.185	0.010	0.833**														
MRT FaceCor	−0.338**	0.153	−0.292**	0.141	0.938**	0.697**													
MRT FaceInCor	0.136	−0.108	−0.068	0.019	0.790**	0.857**	0.749**												
MRT Non-FaceCor	0.153	−0.246*	−0.110	−0.030	0.768**	0.918**	0.726**	0.932**											
MRT Non-FaceInCor	−0.0.133	0.071	−−0.283**	0.088	0.809**	0.781**	0.807**	0.768**	0.772**										
DI	0.519**	0.562**	0.607**	0.068	−0.118	−0.029	−0.160	0.023	−0.099	−0.055									
Age	−0.307**	−0.105	−0.431**	−0.113	0.267**	0.236*	0.163	0.150	0.186	0.085	−0.334**								
Sex	−0.006	−0.089	−0.068	0.164	−0.116	−0.044	−0.152	−0.012	−0.001	−0.078	−0.082	−0.143							
Handedness	−0.035	−0.112	−0.219*	0.061	0.065	0.056	0.057	−0.032	0.032	0.025	−0.136	0.071	−0.123						
Estimated IQ	0.133	0.304**	0.194	0.082	0.145	0.135	0.131	0.205*	0.126	0.272**	0.346**	−0.050	0.010	−0.114					
ADOS social interaction^a^	0.155	−0.113	0.135	0.028	0.172	0.150	0.165	0.284	0.219	0.066	0.130	−0.219	−0.240	0.006	−0.061				
AQ social skills	−0.227*	0.055	−0.146	0.151	0.385**	0.227*	0.382**	0.199	0.200	0.322**	−0.229*	0.058	0.025	0.117	0.202*	−0.186			
AQ attention to details	−0.190	0.033	−0.101	0.128	0.382**	0.254*	0.376**	0.220*	0.232*	0.303**	−0.183	0.071	−0.052	0.041	0.248*	−0.123	0.822**		
AQ attention switching	−0.257*	0.013	−0.188	0.142	0.461**	0.267**	0.465**	0.258*	0.255*	0.377**	−0.301**	0.129	−0.095	0.180	0.103	−0.116	0.877**	0.786**	
AQ total	−0.275**	0.062	−0.189	0.138	0.466**	0.282**	0.466**	0.272**	0.269**	0.391**	−0.256*	0.121	−0.022	0.111	0.199	−0.121	0.934**	0.908**	0.925**
§	% FaceCor	% Non-Face Cor	Post-test % FaceCor	Post-test % Non-FaceCor	MRT Face	MRT Non-Face	MRT FaceCor	MRT FaceInCor	MRT Non-FaceCor	MRT Non-Face InCor	DI	Age	Sex	Handedness	Estimated IQ	ADOS social interaction	AQ social skills	AQ attention to details	AQ attention switching

Cor: correct; MRT: mean RT; DI: discrimination index; IQ: intelligence quotient; ADOS: Autism Diagnostic Observation Schedule; AQ: autism quotient questionnaire.^a^ASD group only.

* *p* < 0.05; **p < 0.01.

For all analyses, values of Cook’s distance were lower than 1 (maximum values per analysis ranged from 0.00 to 0.22). All analyses were repeated with standardized residuals <−3 and >3 removed. Between 0 and 4 cases were excluded to ensure that all residuals fell in the −3 to 3 ranges. Re-analyses did not change the initial outcomes; therefore, data including outliers are reported.

RT in the Face condition was significantly predicted by group (*β* = −0.25, *p* = 0.012) and age (*β* = −0.34, *p* = 0.014), such that individuals with ASD performed worse than controls, and performance declined with increasing age across both groups. There was no significant group-by-age interaction (*β* = 0.036, *p* = 0.79) indicating that the age-related decline in performance was similar in both groups. The total variance explained by the model was 15.7% (*F*_3,92_ = 5.72, *p* = 0.001). None of the other regression analyses showed significant age or behavioral predictors.

#### Navon

The performance on the Navon task is presented in Table 4. There were no significant differences in accuracy on either condition (both *p*’s > 0.17) or local–global precedence differences between groups (*p* = 0.7). However, individuals with ASD were slower on both Global and Local conditions compared to controls (*p*’s < 0.01), and in trials following an error in the same condition (e.g. mistake in Global trial, followed by a Global trial; *p*’s < 0.005).

**Table 4.** Behavioral performance Navon hierarchical letters.

Navon	ASD (*N* = 48)	CTRL (*N* = 48)	Statistics
Accuracy % correct (SD) [range]			
Global	97.5 (31.9) [84 to 100]	96.6 (3.2) [86 to 100]	*F* = 1.9, *p* = 0.17
Local	98.2 (2.7) [83 to 100]	97.7 (1.7) [93 to 100]	*F* = 1.2, *p* = 0.28
RT in ms (SD) [range]			
Global	739.7 (181) [487.7 to 1238.8]	638.8 (102.3) [469.4 to 896.7]	***F* = 11.31, *p* = 0.001**
Local	756.9 (190.1) [498.3 to 1384.7]	660.8 (137.6) [461.1 to 1103.5]	***F* = 8.06, *p* = 0.006**
Post-measures ms (SD) [range]			
Post-correct			
Global	731 (170.7) [487.1 to 1178.1]	633.4 (100) [471.1 to 893]	***F* = 11.68, *p* = 0.001**
Local	749.1 (187.6) [495.6 to 1391.3]	651.3 (133.7) [445.8 to 1110.5]	***F* = 8.65, *p* = 0.004**
Post–error			
Global	960.4 (539.1) [419 to 3200]	818.9 (306.2) [475 to 1955]	*F* = 2.23, *p* = 14
Local	1101.8 (1007.3) [393 to 651]	874.5 (596.2) [537.7 to 4297.7]	*F* = 1.51, *p* = 0.22
Local–Global precedence	17.2 (50.8) [−86.6 to 281.5]	21.8 (66.5) [−59.4 to 281.5]	*F* = 0.15, *p* = 0.70

ASD: autism spectrum disorder; CTRL: control; RT: reaction time.

Numbers in bold reflect significant between-group differences. Outliers are not included in these analyses.

#### Association between holistic face processing and global–local processing

To investigate if differences in holistic face processing (Mooney) can be explained by differences in global–local processing (Navon), we tested the association between performance on the Mooney (scanner version) and the Navon task. There were positive associations between RTs on the Face and Non-Face condition (Mooney) and on the Global and Local condition (Navon; 0.39 < *r*’s < 0.42, *p*’s < 0.001), but not for accuracy. There were no significant differences between the groups in these associations (all *p*’s > 0.1).

### fMRI results

**Figure 3.**
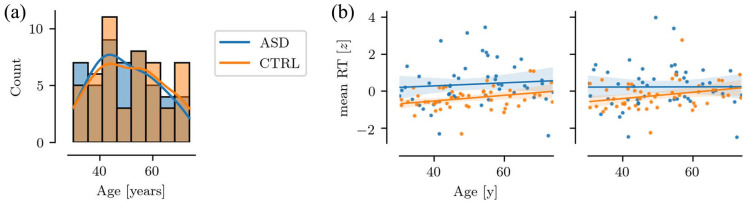
(a) Age distribution in the ASD and CTRL group. (b) Association between age and mean reaction time (mean RT) in the ASD and CTRL group. The face condition is shown on the left and the non-face condition on the right.

**Figure 4.**
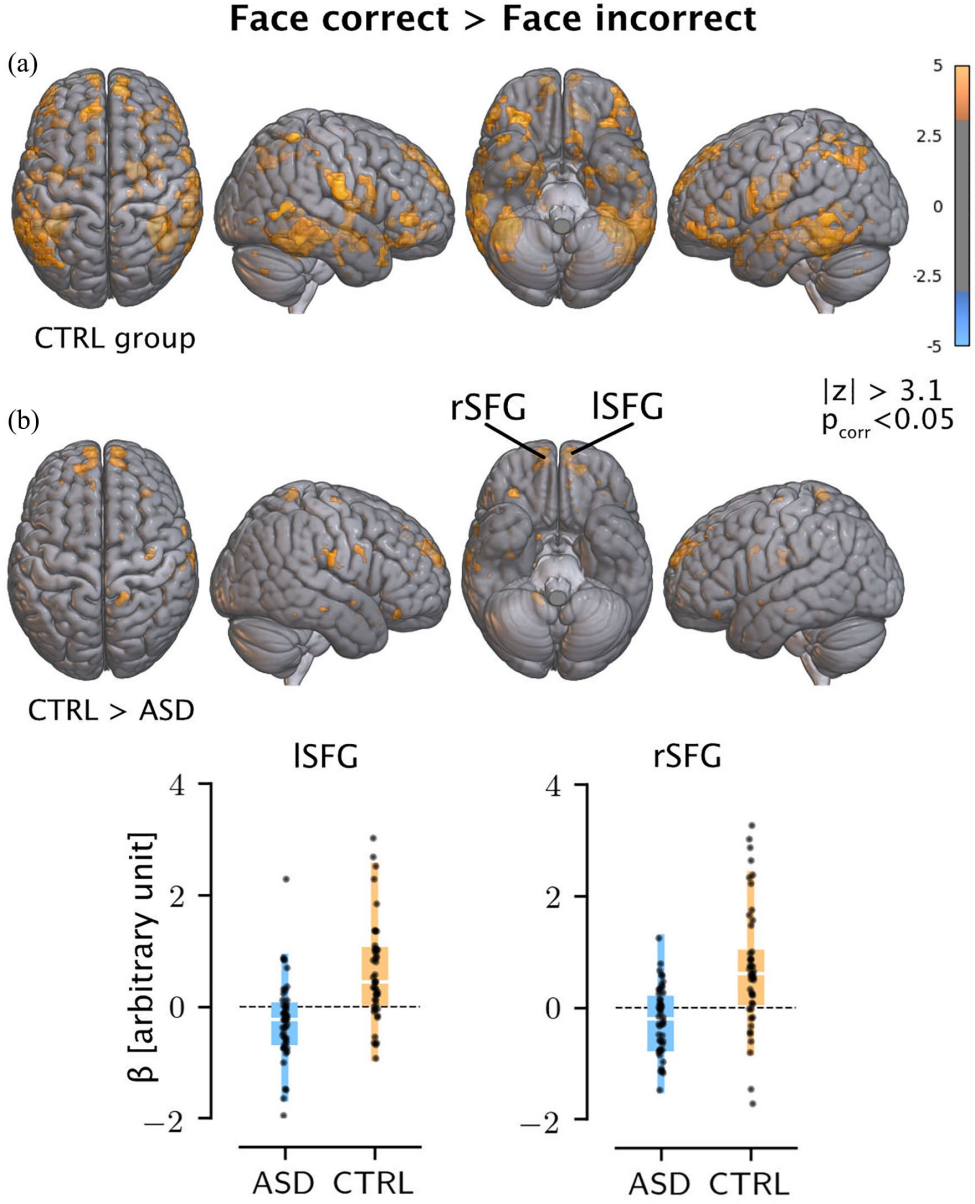
(a) Activation compared to baseline for the CTRL group. (b) Comparison of activation between the ASD and CTRL group. Top panel: results of the comparison of activation in the Face (correct) > Face (incorrect) condition between the ASD and CTRL group. Clusters that survived cluster-level correction for multiple comparisons are shown. Only clusters with a least five voxels were considered (lSFG: left superior frontal gyrus and rSFG: right superior frontal gyrus). Bottom panel: the boxplots show the regression coefficients extracted from the first-level contrasts for the clusters. Each dot represents the value for one participant.

Here, we investigated potential differences in the underlying neural signature of holistic face recognition in ASD. Both groups displayed activation in the core face-processing regions, including in the fusiform gyrus, lateral occipital cortex, and middle and inferior temporal lobe (see Figure 4(a) and Supplementary Table 2 and Supplementary Figure 1). There was a significant group effect for the Face (correct) > Face (incorrect) contrast with lower activation in a left and right superior frontal gyrus (SFG) cluster in the ASD group (left SFG cluster: 8.5, 65.5., 23.8 (*x, y, z*, MNI), M = 4.76 (z), volume = 59.4 (mm^3^); right SFG cluster: −6.5, 65.5, 23.8, M = 4.71, volume = 59.4, see Figure 3(b)). These effects remained when including age as a nuisance regressor. There was no significant group-by-age interaction. There were no significant group or age × group effects for the Face (correct) > Non-Face (correct) contrast.

## Discussion

The aim of this study was to investigate if there are differences in holistic face processing in ASD across the adult lifespan. Even though our results confirm aspects of earlier reported face recognition difficulties in autistic individuals, taken together, we conclude that our data suggest that the differences in holistic face processing are explained by slower processing in this type of task in autistic adults (30–75 years).

The autistic and comparison group both showed age-related slowing on a holistic face cognition task and a global–local processing task. However, autistic adults showed slower RT across all ages and experimental conditions. Furthermore, higher self-reported autistic traits were related to overall slower RTs across face and non-face conditions. Notably, performance increased and reached control levels in autistic adults on the self-paced version of the task. These results argue against a primary face recognition deficit in autistic adults, but point rather to a problem with the pace of information processing. Indeed, reduced processing speed has been repeatedly reported in ASD (Hedvall et al., 2013; Van der Hallen et al., 2015), and has been related to symptom severity (Oliveras-Rentas et al., 2012) and reduced white matter structural integrity (Lazar et al., 2014). Furthermore, slower processing in local–global processing tasks may be exaggerated in older autistic adults (Davids et al., 2020). This slowing may be related to reduced white matter connectivity with aging in older autistic adults that has been previously related to slower cognitive processing (Koolschijn et al., 2017).

For face recognition, our observation of elevated RTs with preserved accuracy is in line with other studies in autistic individuals. For instance, in a configural face change detection study, autistic adults were able to detect both small and large changes but were slower to do so (Faja et al., 2009). Similarly, in a face recognition study, adolescents and adults with ASD showed reduced neural speed of face processing (McPartland et al., 2004). In sum, previous studies and our results suggest that differences in face processing in autism may be tied to reduced processing speed rather than a core problem with face processing per se.

Our results further suggest that slower processing speed influences not only face recognition but also performance on local–global processing tasks. The Navon task results show neither enhanced local visual processing nor a deficit in global visual processing. However, we did find elevated RTs in ASD irrespective of condition. Moreover, RTs were highly correlated between the face-processing and local–global processing tasks, which suggest a general slower processing speed in ASD. These findings on the Navon task are in line with studies that reported elevated RTs for face recognition but no global processing problems (e.g. Behrmann et al., 2006; Guy et al., 2019; Hedley et al., 2011, 2012; Nishimura et al., 2008). We conclude that there is no severe impairment in holistic processing and global–local processing as we found that autistic individuals were only less accurate in identifying faces when they did not have sufficient time.

Our fMRI results further support the interpretation that there is no specific face-processing deficit in autistic adults, at least when processing Mooney faces. There were no significant differences within regions of the core face-processing network, that is, fusiform gyrus, and inferior frontal, occipital and temporal regions (Ammons et al., 2020; Andrews & Schluppeck, 2004; Grutzner et al., 2010). In conjunction with the group-activation maps, this suggests that the same face-processing regions are recruited in autistic individuals when processing Mooney faces. These results are in line with other studies reporting no differences in activation patterns between faces and objects (e.g. Bird et al., 2006; Kleinhans et al., 2008), or for the fusiform gyrus specifically (Hadjikhani et al., 2004; Pierce et al., 2001). In contrast, our results indicated lower activation in the bilateral SFG in the autism group. The SFG has been implicated in a variety of functions, including working memory (Courtney et al., 1998; Owen et al., 1996; Rowe et al., 2000) and cognitive control (Andrews-Hanna et al., 2014; Niendam et al., 2012; Raichle, 2015; Vincent et al., 2008). The SFG sits at the intersection of several major anatomical and functional networks (Briggs et al., 2020; Li et al., 2013), including the fronto-parietal attention network and the default mode network. Reduced connectivity in both networks and of the SFG have been repeatedly observed in autistic individuals (see Hull et al., 2017, for a review). Together with our behavioral results, the neuroimaging findings may suggest differences in general cognitive processing rather than specific face-processing differences in autistic adults (30–75 years). Future studies with imaging modalities that are more sensitive to the speed of neural processing, that is, M/EEG, may provide further mechanistic insight into the interaction between processing speed and face processing in autism. Furthermore, future studies should investigate if similar results are observed with more ecologically valid face stimuli.

The study has some potential limitations. While the current sample is relatively large compared to other fMRI studies including autistic adults, an issue can be raised concerning the extent to which the sample represents the general autistic population, as this is the first fMRI study of face processing that includes older adults without intellectual impairment. Based on the relatively low AQ and ADOS scores and the adulthood diagnoses, some may argue that our sample only represents autistic individuals with relatively mild challenges, for example, see also prior discussions on this topic (Koolschijn et al., 2017). While autistic traits as measured with the AQ do not seem to correlate with age (Lodi-Smith et al., 2021), the psychometric properties of AQ and ADOS in older adults have been questioned (Baghdadli et al., 2017; Bastiaansen et al., 2011). Nevertheless, our mean AQ scores match those from other ASD studies (Blomqvist et al., 2014; Kirkovski et al., 2015; Mueller et al., 2013; Ring et al., 2020; Roine et al., 2013; Ruzich et al., 2015; Yasuda et al., 2014). In sum, our findings may not generalize to the full autism spectrum, particularly to those with intellectual impairment. However, please note that IQ did not have a significant influence in either task in our sample. Another limitation is the use of Mooney faces. The Mooney faces were designed to tap primary holistic face processing. However, processing these abstract stimuli may not be representative of processing real-world faces that are dynamic and rich in detail. Furthermore, the use of scrambled Mooney faces as control stimuli in this study was not ideal. The scrambled Mooney faces contain high-frequency content and sharp edges that make the task easier. Future work should use inverted Mooney faces as control stimuli to remove these confounds.

## Conclusion

In this study, we set out to test if holistic face-processing difficulties are present in autistic adults across the adult lifespan. Our results indicate that holistic face processing is not different compared to typical controls of the same age. Furthermore, we find no differences in core face-processing networks in an fMRI task of holistic face processing. Instead, our findings suggest slower information processing in autistic adults across holistic face-processing and local–global processing tasks. Our neuroimaging results further suggest differences in neural substrates that are implicated in cognitive control and working memory.

## Supplemental Material

sj-docx-1-aut-10.1177_13623613211065297 – Supplemental material for Atypically slow processing of faces and non-faces in older autistic adults Click here for additional data file. Supplemental material, sj-docx-1-aut-10.1177_13623613211065297 for Atypically slow processing of faces and non-faces in older autistic adults by Joe Bathelt, P Cédric MP Koolschijn and Hilde M Geurts in Autism

## Notes

1 We use the term autism spectrum disorder (ASD) here to apply the same nomenclature as the *Diagnostic and Statistical Manual of Mental Disorders* (5th ed.; DSM-5). This does not imply that we endorse the description of autism as a disorder. We, therefore, also intermix the use of the term ASD with identity first language.

2 Please note that clinicians have to follow national guidelines in the country where this study was conducted. These guidelines necessitate the assessment of developmental history in childhood for an ASD diagnosis in adulthood.
