# Supplementary material for: Atypically slow processing of faces and non-faces in older autistic adults
Source: Autism. 2021 Dec 28;26(7):1737–51. doi: 10.1177/13623613211065297 (PMC9483195; doi:10.1177/13623613211065297)
Supplement: sj-docx-1-aut-10.1177_13623613211065297 – Supplemental material for Atypically slow processing of faces and non-faces in older autistic adults [file sj-docx-1-aut-10.1177_13623613211065297.docx]

**Suppl Table 1. Additional demographic variables**

| **Description** | **ASD** | **CTRL** | **Statistics** |
| --- | --- | --- | --- |
|  | N=50 | N=49 |  |
| Barthel total score | 19.84 (.37)[19-20] | 19.94 (.24)[19-20] | *F*=2.46, *p*=.120 |
| Currently smoking | 9 (18%) | 2 (4%) | ***χ^2^*=4.85, *p*=.028** |
| Active in physical exercise^a^ | 31 (62%) | 42 (86%) | ***χ^2^*=7.19, *p*=.007** |
| MINI current |  |  |  |
| Depression | 1 (2%) | 1 (2%) | *χ^2^*<.001, *p*=.99 |
| Dysthymia | 2 (4%) 2 missing | 0 | *χ^2^*=4.90, *p*=.130 |
| Mania | 1 (2%) | 0 | *χ^2^*=.99, *p*=.320 |
| Hypomania | 2 (4%) 1 missing | 0 | *χ^2^*=3.03, *p*=.220 |
| Panic disorder | 2 (4%) | 1 (2%) | *χ^2^*=.32, *p*=.570 |
| Agoraphobia | 11 (22%) | 0 | ***χ^2^*=12.13, *p<*.001** |
| Social phobia | 5 (10%) 4 missing | 1 (2%) | ***χ^2^*=7.21, *p*=.027** |
| Single phobia | 4 (8%) | 1 (2%) | ***χ^2^***=1.83, *p*=.176 |
| Obsessive compulsive symptoms | 6 (12%), 7 missing | 1 (2%) | ***χ^2^*=11.99, *p*=.002** |
| PTSD | 2 (4%), 1 missing | 0 | *χ^2^*=3.03, *p*=.220 |
| Alcohol dependency | 1 (2%) | 0 | *χ^2^*=.99, *p*=.320 |
| Alcohol abuse | 1 (2%), 6 missing | 1 (2%) 3 missing | *χ^2^*=1.04, *p*=.596 |
| Substance abuse | 0 | 0 (1 missing) | *χ^2^*=.002, *p*=.967 |
| Bulimia nervosa | 1 (2%) | 0, 1 missing | *χ^2^*=2.00, *p*=.368 |
| Generalized anxiety disorder | 5 (10%), 3 missing | 1 (2%) 1 missing | *χ^2^*=3.94, *p*=.140 |
| Premenstrual dysphoria | 0 | 1 (2%) | *χ^2^*=1.55, *p*=.463 |
| Note. Numbers in bold reflect significant between group differences.  ^a^ Defined as any type of physical exercise independent of duration  Abbreviations: MoCA, Montreal Cognitive Assessment; MMSE, Mini-mental state examination; MINI, Mini International Neuropsychiatric Interview; PTSD, Posttraumatic stress disorder. | | | |

**Suppl Table 2. Significant clusters compared to baseline for the Face(correct)>Face(incorrect) contrast with the groups pooled together.**

|  | **Peak [MNI coordinates]** | | |  | |  | |  | |  |
| --- | --- | --- | --- | --- | --- | --- | --- | --- | --- | --- |
| **cluster** | **x** | **y** | **z** | | **mean [z]** | | **Volume [mm]** | | **DK atlas labels** | |
| 1 | -39.5 | -45.5 | -19.1 | | 3.87 | | 16840 | | 30.16% Unknown; 22.40% ctx-lh-inferiortemporal; 17.11% Left-Cerebral-White-Matter; 16.58% ctx-lh-fusiform;  10.05% ctx-lh-middletemporal | |
| 2 | 41.5 | -48.5 | -19.1 | | 3.97 | | 16008 | | 27.27% Unknown; 23.38% Right-Cerebral-White-Matter; 21.52% ctx-rh-fusiform; 17.25% ctx-rh-middletemporal;  6.49% ctx-rh-inferiortemporal | |
| 3 | -51.5 | -54.5 | 56.8 | | 3.50 | | 14672 | | 41.70% Unknown; 21.66% Left-Cerebral-White-Matter; 15.59% ctx-lh-inferiorparietal; 11.54% ctx-lh-supramarginal;  7.49% ctx-lh-postcentral | |
| 4 | -45.5 | 47.5 | -12.5 | | 3.42 | | 6237 | | 44.76% Unknown; 23.81% ctx-lh-parstriangularis; 13.33% ctx-lh-parsorbitalis; 8.10% ctx-lh-rostralmiddlefrontal;  8.10% Left-Cerebral-White-Matter | |
| 5 | 47.5 | 38.5 | 0.7 | | 3.57 | | 4158 | | 54.29% ctx-rh-parstriangularis; 27.86% Unknown; 16.43% Right-Cerebral-White-Matter | |
| 6 | -21.5 | -3.5 | -22.4 | | 3.48 | | 3920 | | 37.12% Left-Amygdala; 12.88% Left-Hippocampus; 12.88% Left-Cerebral-White-Matter; 10.61% Unknown;  9.09% Left-VentralDC; 6.82% Left-Putamen; 5.30% ctx-lh-medialorbitofrontal | |
| 7 | 26.5 | -0.5 | -19.1 | | 3.47 | | 3208 | | 46.30% Right-Amygdala; 12.96% Unknown; 11.11% ctx-rh-insula; 10.19% Right-Putamen; 9.26% Right-Cerebral-White-Matter;  5.56% ctx-rh-unknown | |
| 8 | -39.5 | -3.5 | 13.9 | | 3.59 | | 2465 | | 34.94% Unknown; 22.89% Left-Putamen; 18.07% ctx-lh-insula; 16.87% Left-Cerebral-White-Matter | |
| 9 | 44.5 | -48.5 | 56.8 | | 3.47 | | 2228 | | 46.67% Unknown; 29.33% ctx-rh-inferiorparietal; 14.67% ctx-rh-superiorparietal; 9.33% Right-Cerebral-White-Matter | |
| 10 | 38.5 | -72.5 | 50.2 | | 3.30 | | 1782 | | 41.67% ctx-rh-inferiorparietal; 30.00% Unknown; 28.33% Right-Cerebral-White-Matter | |
| 11 | -66.5 | -15.5 | -12.5 | | 3.29 | | 1455 | | 55.10% ctx-lh-middletemporal; 28.57% Unknown; 16.33% Left-Cerebral-White-Matter | |
| 12 | 35.5 | 41.5 | -12.5 | | 3.45 | | 1158 | | 30.77% Unknown; 28.21% ctx-rh-lateralorbitofrontal; 25.64% ctx-rh-parsorbitalis; 15.38% Right-Cerebral-White-Matter | |
| 13 | -30.5 | 20.5 | 56.8 | | 3.32 | | 950 | | 50.00% ctx-lh-caudalmiddlefrontal; 34.38% Unknown; 15.62% Left-Cerebral-White-Matter | |
| 14 | 8.5 | 29.5 | 33.7 | | -3.35 | | 921 | | 61.29% Unknown; 19.35% ctx-lh-superiorfrontal; 12.90% ctx-lh-caudalanteriorcingulate; 6.45% ctx-rh-superiorfrontal | |
| 15 | 50.5 | -78.5 | -2.6 | | 3.50 | | 832 | | 50.00% Unknown; 46.43% ctx-rh-lateraloccipital | |
| 16 | 26.5 | -3.5 | 7.3 | | 3.42 | | 713 | | 79.17% Right-Putamen; 16.67% Right-Cerebral-White-Matter | |
| 17 | 26.5 | 53.5 | 20.5 | | -3.21 | | 683 | | 43.48% Unknown; 30.43% Right-Cerebral-White-Matter; 26.09% ctx-rh-rostralmiddlefrontal | |
| 18 | -15.5 | 41.5 | 50.2 | | 3.27 | | 653 | | 50.00% ctx-lh-superiorfrontal; 27.27% Left-Cerebral-White-Matter; 22.73% Unknown | |
| 19 | -54.5 | 8.5 | 7.3 | | 3.22 | | 624 | | 47.62% ctx-lh-precentral; 33.33% Unknown; 19.05% Left-Cerebral-White-Matter | |
| 20 | 68.5 | -9.5 | 20.5 | | 3.32 | | 594 | | 70.00% ctx-rh-postcentral; 25.00% Unknown; 5.00% Right-Cerebral-White-Matter | |
| 21 | 50.5 | 2.5 | 23.8 | | 3.35 | | 594 | | 45.00% Right-Cerebral-White-Matter; 35.00% ctx-rh-precentral; 15.00% Unknown; 5.00% ctx-rh-parsopercularis | |
| 22 | -21.5 | -63.5 | 13.9 | | -3.23 | | 564 | | 42.11% ctx-lh-precuneus; 31.58% Unknown; 26.32% Left-Cerebral-White-Matter | |
| 23 | -0.5 | 5.5 | 60.1 | | -3.34 | | 446 | | 86.67% Unknown; 13.33% ctx-lh-superiorfrontal | |
| 24 | 41.5 | 50.5 | -5.9 | | 3.23 | | 416 | | 50.00% ctx-rh-parsorbitalis; 42.86% Right-Cerebral-White-Matter; 7.14% Unknown | |
| 25 | 41.5 | -0.5 | 10.6 | | 3.33 | | 386 | | 69.23% Unknown; 30.77% ctx-rh-precentral | |
| 26 | -3.5 | -33.5 | 37.0 | | 3.18 | | 386 | | 76.92% Unknown; 7.69% ctx-rh-isthmuscingulate; 7.69% ctx-lh-precuneus; 7.69% ctx-lh-posteriorcingulate | |
| 27 | 17.5 | -21.5 | -9.2 | | 3.22 | | 386 | | 46.15% Unknown; 15.38% ctx-rh-parahippocampal; 15.38% Right-Hippocampus; 15.38% Right-Cerebral-White-Matter;  7.69% Brain-Stem | |
| 28 | 35.5 | -69.5 | -42.2 | | 3.25 | | 356 | | 100.00% Right-Cerebellum-Cortex | |
| 29 | 41.5 | -12.5 | -32.3 | | 3.40 | | 297 | | 60.00% Unknown; 30.00% ctx-rh-fusiform; 10.00% Right-Cerebral-White-Matter | |
| 30 | 41.5 | 20.5 | -32.3 | | 3.52 | | 297 | | 30.00% ctx-rh-temporalpole; 30.00% ctx-rh-superiortemporal; 30.00% Right-Cerebral-White-Matter;  10.00% ctx-rh-middletemporal | |
| 31 | -9.5 | 17.5 | -2.6 | | 3.32 | | 297 | | 60.00% Left-Caudate; 30.00% Left-Accumbens-area; 10.00% Left-Cerebral-White-Matter | |
| 32 | -57.5 | 8.5 | 27.1 | | 3.26 | | 267 | | 55.56% ctx-lh-precentral; 22.22% Left-Cerebral-White-Matter; 22.22% Unknown | |
| 33 | -57.5 | -15.5 | 17.2 | | 3.24 | | 238 | | 50.00% ctx-lh-postcentral; 25.00% ctx-lh-supramarginal; 12.50% Left-Cerebral-White-Matter; 12.50% Unknown | |
| 34 | -27.5 | 32.5 | -22.4 | | 3.18 | | 208 | | 71.43% Unknown; 28.57% ctx-lh-lateralorbitofrontal | |
| 35 | -3.5 | 20.5 | -12.5 | | 3.17 | | 178 | | 83.33% ctx-lh-medialorbitofrontal; 16.67% Unknown | |
| 36 | 17.5 | -57.5 | -19.1 | | 3.07 | | 178 | | 100.00% Right-Cerebellum-Cortex | |
| 37 | 20.5 | -15.5 | -25.7 | | 3.24 | | 149 | | 40.00% ctx-rh-unknown; 20.00% ctx-rh-parahippocampal; 20.00% ctx-rh-entorhinal; 20.00% Right-Hippocampus | |
| 38 | 56.5 | 11.5 | 13.9 | | 3.16 | | 149 | | 60.00% ctx-rh-precentral; 40.00% Unknown | |
| 39 | -42.5 | 17.5 | 37.0 | | 3.09 | | 119 | | 50.00% ctx-lh-caudalmiddlefrontal; 50.00% Left-Cerebral-White-Matter | |
| 40 | -12.5 | 11.5 | -12.5 | | 3.07 | | 119 | | 75.00% Left-Cerebral-White-Matter; 25.00% Left-Putamen | |
| 41 | 8.5 | 59.5 | 33.7 | | 3.17 | | 119 | | 75.00% ctx-rh-superiorfrontal; 25.00% Right-Cerebral-White-Matter | |
| 42 | 11.5 | -78.5 | -29.0 | | 3.06 | | 119 | | 100.00% Right-Cerebellum-Cortex | |
| 43 | 23.5 | -66.5 | -2.6 | | -3.08 | | 89 | | 100.00% Right-Cerebral-White-Matter | |
| 44 | -33.5 | -12.5 | -22.4 | | 3.04 | | 89 | | 100.00% Left-Hippocampus | |
| 45 | -21.5 | -72.5 | 27.1 | | -3.08 | | 89 | | 100.00% Left-Cerebral-White-Matter | |
| 46 | -3.5 | 14.5 | 43.6 | | -3.20 | | 89 | | 100.00% ctx-lh-superiorfrontal | |
| 47 | -15.5 | 5.5 | 10.6 | | 3.10 | | 89 | | 66.67% Left-Caudate; 33.33% Left-Cerebral-White-Matter | |
| 48 | -30.5 | -54.5 | 56.8 | | 3.08 | | 59 | | 100.00% Left-Cerebral-White-Matter | |
| 49 | -45.5 | -45.5 | 4.0 | | 3.10 | | 59 | | 100.00% Unknown | |
| 50 | 32.5 | -48.5 | -2.6 | | -3.21 | | 59 | | 100.00% Right-Cerebral-White-Matter | |
| 51 | 29.5 | -9.5 | -38.9 | | 3.31 | | 59 | | 100.00% ctx-rh-fusiform | |
| 52 | -12.5 | 8.5 | 7.3 | | 3.17 | | 59 | | 50.00% Left-Caudate; 50.00% Left-Cerebral-White-Matter | |
| 53 | -6.5 | 26.5 | -19.1 | | 3.46 | | 59 | | 100.00% ctx-lh-medialorbitofrontal | |
| 54 | 14.5 | 11.5 | 63.4 | | -3.03 | | 59 | | 50.00% ctx-rh-superiorfrontal; 50.00% Right-Cerebral-White-Matter | |
| 55 | 17.5 | 11.5 | 60.1 | | -3.08 | | 59 | | 50.00% ctx-rh-superiorfrontal; 50.00% Right-Cerebral-White-Matter | |
| 56 | 26.5 | 17.5 | -22.4 | | 3.01 | | 59 | | 100.00% ctx-rh-lateralorbitofrontal | |
| 57 | 29.5 | -57.5 | 70.0 | | 3.01 | | 59 | | 100.00% Unknown | |
| 58 | -27.5 | -72.5 | -5.9 | | -3.12 | | 59 | | 50.00% ctx-lh-fusiform; 50.00% Left-Cerebral-White-Matter | |
| 59 | 35.5 | -72.5 | -45.5 | | 3.08 | | 30 | | 100.00% Right-Cerebellum-Cortex | |
| 60 | -24.5 | -54.5 | 4.0 | | -3.01 | | 30 | | 100.00% Left-Cerebral-White-Matter | |
| 61 | 59.5 | -66.5 | 17.2 | | 3.06 | | 30 | | 100.00% Unknown | |
| 62 | -60.5 | 5.5 | 23.8 | | 3.15 | | 30 | | 100.00% ctx-lh-precentral | |
| 63 | 47.5 | 47.5 | -12.5 | | 3.00 | | 30 | | 100.00% ctx-rh-parsorbitalis | |
| 64 | -12.5 | -78.5 | -38.9 | | 3.23 | | 30 | | 100.00% Left-Cerebellum-White-Matter | |
| 65 | 32.5 | -72.5 | -42.2 | | 3.23 | | 30 | | 100.00% Right-Cerebellum-Cortex | |
| 66 | -48.5 | -42.5 | 7.3 | | 3.02 | | 30 | | 100.00% ctx-lh-bankssts | |
| 67 | -12.5 | -30.5 | -2.6 | | 3.08 | | 30 | | 100.00% Left-Thalamus-Proper | |
| 68 | -45.5 | 14.5 | -35.6 | | 3.44 | | 30 | | 100.00% ctx-lh-middletemporal | |
| 69 | 29.5 | -75.5 | 56.8 | | 3.00 | | 30 | | 100.00% Unknown | |
| 70 | 17.5 | -18.5 | -19.1 | | 3.10 | | 30 | | 100.00% Unknown | |
| 71 | 17.5 | 44.5 | 43.6 | | 3.00 | | 30 | | 100.00% ctx-rh-superiorfrontal | |

**Figure S1: Overview of the clusters in the Face(correct)>Face(incorrect) contrast compared to baseline with both groups pooled together.**

**
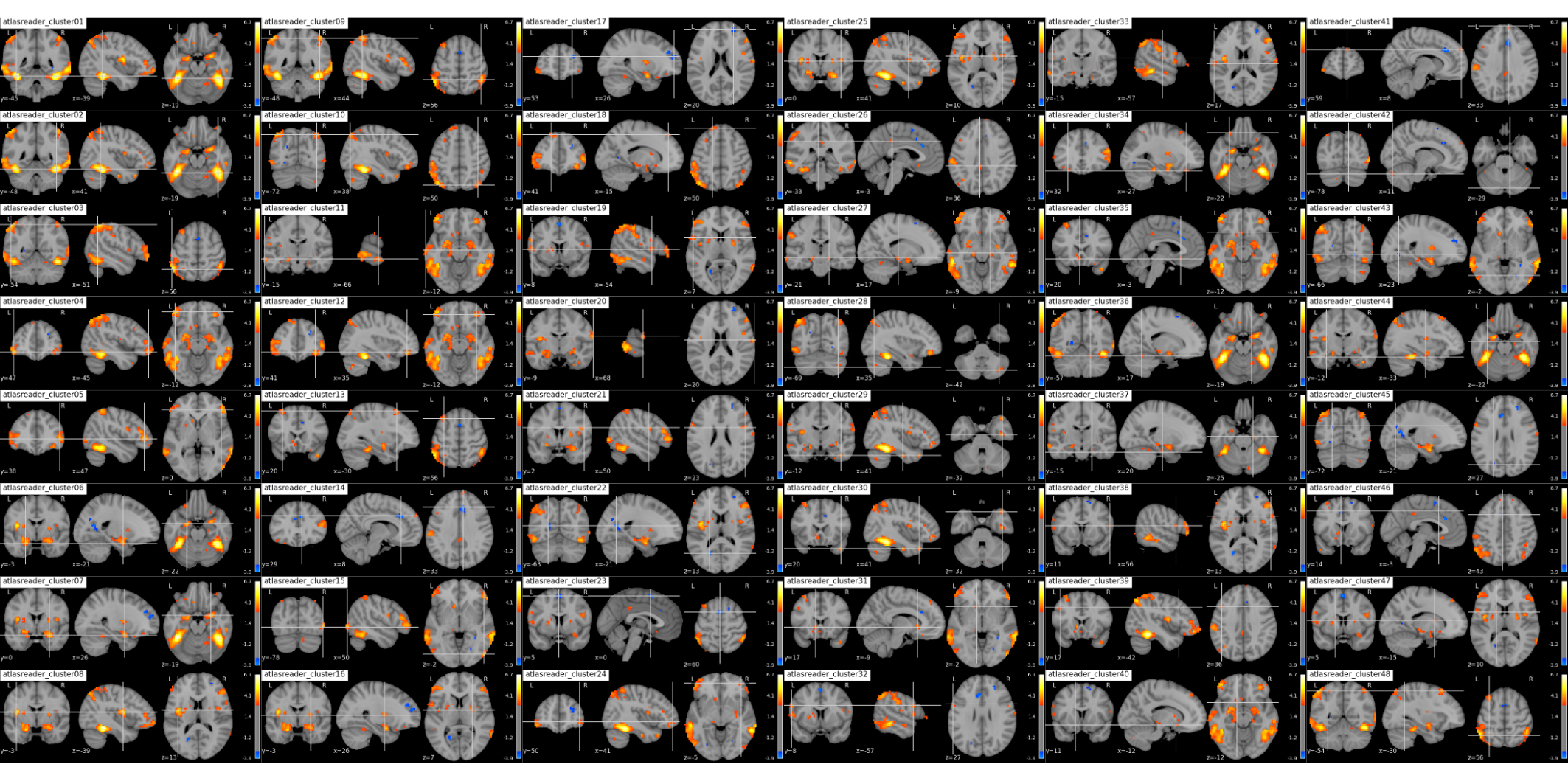
**

**
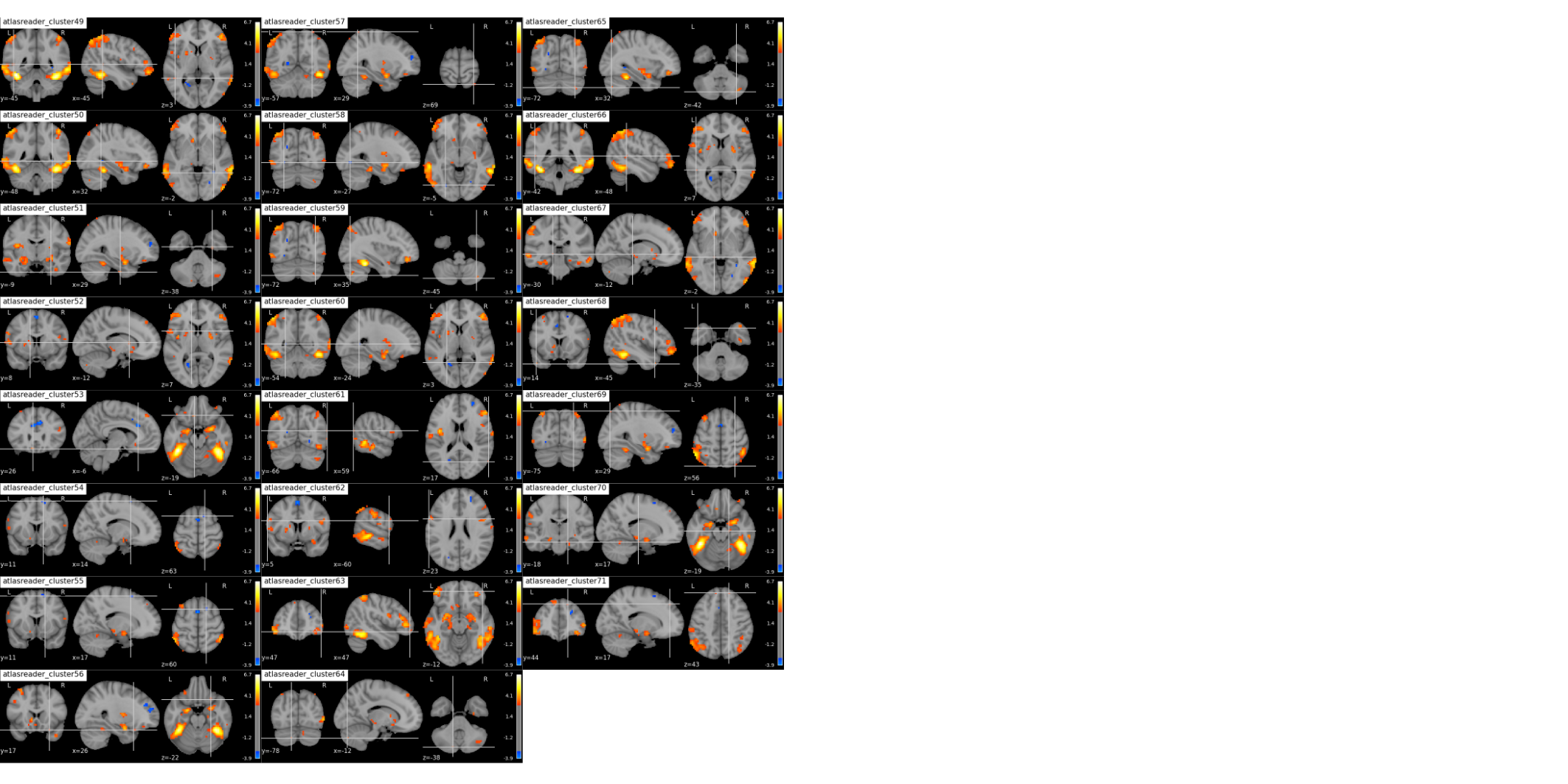
**

**Suppl Methods**

The following description was automatically generated by fMRIPrep v 20.1.1 1 (Esteban, Markiewicz, et al. (2018); Esteban, Blair, et al. (2018); RRID:SCR_016216) and is included here to make the pre-processing procedure transparent and reproducible.

**Anatomical data preprocessing:** The T1-weighted (T1w) image was corrected for intensity non-uniformity (INU) with N4BiasFieldCorrection (Tustison et al. 2010), distributed with ANTs 2.2.0 (Avants et al. 2008, RRID:SCR_004757), and used as T1w-reference throughout the workflow. The T1w-reference was then skull-stripped with a *Nipype* implementation of the antsBrainExtraction.sh workflow (from ANTs), using OASIS30ANTs as the target template. Brain tissue segmentation of cerebrospinal fluid (CSF), white-matter (WM) and gray-matter (GM) was performed on the brain-extracted T1w using fast (FSL 5.0.9, RRID:SCR_002823, Zhang, Brady, and Smith 2001). Brain surfaces were reconstructed using recon-all (FreeSurfer 6.0.1, RRID:SCR_001847, Dale, Fischl, and Sereno 1999), and the brain mask estimated previously was refined with a custom variation of the method to reconcile ANTs-derived and FreeSurfer-derived segmentations of the cortical gray-matter of Mindboggle (RRID:SCR_002438, Klein et al. 2017). Volume-based spatial normalization to one standard space (MNI152NLin2009cAsym) was performed through nonlinear registration with antsRegistration (ANTs 2.2.0), using brain-extracted versions of both T1w reference and the T1w template. The following template was selected for spatial normalization: *ICBM 152 Nonlinear Asymmetrical template version 2009c* [Fonov et al. (2009), RRID:SCR_008796; TemplateFlow ID: MNI152NLin2009cAsym].

**Functional data preprocessing:** For each of the 2 BOLD runs per subject, the following preprocessing was performed. First, a reference volume and its skull-stripped version were generated using a custom methodology of *fMRIPrep*. Head-motion parameters with respect to the BOLD reference (transformation matrices, and six corresponding rotation and translation parameters) are estimated before any spatiotemporal filtering using mcflirt (FSL 5.0.9, Jenkinson et al. 2002). Susceptibility distortion correction (SDC) was omitted. The BOLD reference was then co-registered to the T1w reference using bbregister (FreeSurfer) which implements boundary-based registration (Greve and Fischl 2009). Co-registration was configured with six degrees of freedom. The BOLD time-series (including slice-timing correction when applied) were resampled onto their original, native space by applying the transforms to correct for head-motion. These resampled BOLD time-series will be referred to as *preprocessed BOLD in original space*, or just *preprocessed BOLD*. The BOLD time-series were resampled into standard space, generating a *preprocessed BOLD run in MNI152NLin2009cAsym space*. First, a reference volume and its skull-stripped version were generated using a custom methodology of *fMRIPrep*. Several confounding time-series were calculated based on the *preprocessed BOLD*: framewise displacement (FD), DVARS and three region-wise global signals. FD was computed using two formulations following Power (absolute sum of relative motions, Power et al. (2014)) and Jenkinson (relative root mean square displacement between affines, Jenkinson et al. (2002)). FD and DVARS are calculated for each functional run, both using their implementations in *Nipype* (following the definitions by Power et al. 2014). The head-motion estimates calculated in the correction step were also placed within the corresponding confounds file. The confound time series derived from head motion estimates and global signals were expanded with the inclusion of temporal derivatives and quadratic terms for each (Satterthwaite et al. 2013). Frames that exceeded a threshold of 0.5 mm FD or 1.5 standardised DVARS were annotated as motion outliers. All resamplings can be performed with *a single interpolation step* by composing all the pertinent transformations (i.e. head-motion transform matrices, susceptibility distortion correction when available, and co-registrations to anatomical and output spaces). Gridded (volumetric) resamplings were performed using antsApplyTransforms (ANTs), configured with Lanczos interpolation to minimize the smoothing effects of other kernels (Lanczos 1964). Non-gridded (surface) resamplings were performed using mri_vol2surf (FreeSurfer).

Many internal operations of *fMRIPrep* use *Nilearn* 0.6.2 (Abraham et al. 2014, RRID:SCR_001362), mostly within the functional processing workflow. For more details of the pipeline, see [the section corresponding to workflows in *fMRIPrep*’s documentation](https://fmriprep.readthedocs.io/en/latest/workflows.html).

**References**

Abraham, Alexandre, Fabian Pedregosa, Michael Eickenberg, Philippe Gervais, Andreas Mueller, Jean Kossaifi, Alexandre Gramfort, Bertrand Thirion, and Gael Varoquaux. 2014. “Machine Learning for Neuroimaging with Scikit-Learn.” *Frontiers in Neuroinformatics* 8. <https://doi.org/10.3389/fninf.2014.00014>.

Avants, B.B., C.L. Epstein, M. Grossman, and J.C. Gee. 2008. “Symmetric Diffeomorphic Image Registration with Cross-Correlation: Evaluating Automated Labeling of Elderly and Neurodegenerative Brain.” *Medical Image Analysis* 12 (1): 26–41. <https://doi.org/10.1016/j.media.2007.06.004>.

Dale, Anders M., Bruce Fischl, and Martin I. Sereno. 1999. “Cortical Surface-Based Analysis: I. Segmentation and Surface Reconstruction.” *NeuroImage* 9 (2): 179–94. <https://doi.org/10.1006/nimg.1998.0395>.

Esteban, Oscar, Ross Blair, Christopher J. Markiewicz, Shoshana L. Berleant, Craig Moodie, Feilong Ma, Ayse Ilkay Isik, et al. 2018. “FMRIPrep.” *Software*. Zenodo. <https://doi.org/10.5281/zenodo.852659>.

Esteban, Oscar, Christopher Markiewicz, Ross W Blair, Craig Moodie, Ayse Ilkay Isik, Asier Erramuzpe Aliaga, James Kent, et al. 2018. “fMRIPrep: A Robust Preprocessing Pipeline for Functional MRI.” *Nature Methods*. <https://doi.org/10.1038/s41592-018-0235-4>.

Fonov, VS, AC Evans, RC McKinstry, CR Almli, and DL Collins. 2009. “Unbiased Nonlinear Average Age-Appropriate Brain Templates from Birth to Adulthood.” *NeuroImage* 47, Supplement 1: S102. <https://doi.org/10.1016/S1053-8119(09)70884-5>.

Gorgolewski, K., C. D. Burns, C. Madison, D. Clark, Y. O. Halchenko, M. L. Waskom, and S. Ghosh. 2011. “Nipype: A Flexible, Lightweight and Extensible Neuroimaging Data Processing Framework in Python.” *Frontiers in Neuroinformatics*5: 13. <https://doi.org/10.3389/fninf.2011.00013>.

Gorgolewski, Krzysztof J., Oscar Esteban, Christopher J. Markiewicz, Erik Ziegler, David Gage Ellis, Michael Philipp Notter, Dorota Jarecka, et al. 2018. “Nipype.” *Software*. Zenodo. <https://doi.org/10.5281/zenodo.596855>.

Greve, Douglas N, and Bruce Fischl. 2009. “Accurate and Robust Brain Image Alignment Using Boundary-Based Registration.” *NeuroImage* 48 (1): 63–72. <https://doi.org/10.1016/j.neuroimage.2009.06.060>.

Jenkinson, Mark, Peter Bannister, Michael Brady, and Stephen Smith. 2002. “Improved Optimization for the Robust and Accurate Linear Registration and Motion Correction of Brain Images.” *NeuroImage* 17 (2): 825–41. <https://doi.org/10.1006/nimg.2002.1132>.

Klein, Arno, Satrajit S. Ghosh, Forrest S. Bao, Joachim Giard, Yrjö Häme, Eliezer Stavsky, Noah Lee, et al. 2017. “Mindboggling Morphometry of Human Brains.” *PLOS Computational Biology* 13 (2): e1005350. <https://doi.org/10.1371/journal.pcbi.1005350>.

Lanczos, C. 1964. “Evaluation of Noisy Data.” *Journal of the Society for Industrial and Applied Mathematics Series B Numerical Analysis* 1 (1): 76–85. <https://doi.org/10.1137/0701007>.

Power, Jonathan D., Anish Mitra, Timothy O. Laumann, Abraham Z. Snyder, Bradley L. Schlaggar, and Steven E. Petersen. 2014. “Methods to Detect, Characterize, and Remove Motion Artifact in Resting State fMRI.” *NeuroImage* 84 (Supplement C): 320–41. <https://doi.org/10.1016/j.neuroimage.2013.08.048>.

Satterthwaite, Theodore D., Mark A. Elliott, Raphael T. Gerraty, Kosha Ruparel, James Loughead, Monica E. Calkins, Simon B. Eickhoff, et al. 2013. “An improved framework for confound regression and filtering for control of motion artifact in the preprocessing of resting-state functional connectivity data.” *NeuroImage* 64 (1): 240–56. <https://doi.org/10.1016/j.neuroimage.2012.08.052>.

Tustison, N. J., B. B. Avants, P. A. Cook, Y. Zheng, A. Egan, P. A. Yushkevich, and J. C. Gee. 2010. “N4ITK: Improved N3 Bias Correction.” *IEEE Transactions on Medical Imaging* 29 (6): 1310–20. <https://doi.org/10.1109/TMI.2010.2046908>.

Zhang, Y., M. Brady, and S. Smith. 2001. “Segmentation of Brain MR Images Through a Hidden Markov Random Field Model and the Expectation-Maximization Algorithm.” *IEEE Transactions on Medical Imaging* 20 (1): 45–57. <https://doi.org/10.1109/42.906424>.
